# How Can Students’ Entrepreneurial Intention Be Increased? The Role of Psychological Capital, Perceived Learning From an Entrepreneurship Education Program, Emotions and Their Relationships

**DOI:** 10.5964/ejop.2889

**Published:** 2022-02-25

**Authors:** Séverine Chevalier, Isabelle Calmé, Hélène Coillot, Karine Le Rudulier, Evelyne Fouquereau

**Affiliations:** 1 EE 1901, Work and Organizational Psychology, University of Tours, Tours, France; 2 EA 6296, Management Science, University of Tours, Tours, France; 3 UMR CNRS 6262, Management Science, University of Rennes 1, Rennes, France; Connection Lab, San Francisco, CA, USA

**Keywords:** psychological capital, entrepreneurial intention, entrepreneurship education

## Abstract

Entrepreneurship education has become a major focus of interest for researchers and national policy makers to encourage students to pursue entrepreneurial careers. The research on entrepreneurship education–entrepreneurial intentions (EIs) has yielded mixed results, and indicates the need to focus on antecedents of EI. More precisely, the aim of this paper was to examine antecedents of students’ EI in French entrepreneurship education programs. Participants were 460 French university undergraduates. Structural equation modeling results revealed that students’ Psychological Capital (PsyCap) had a significant positive relationship with perceived learning from the program and a significant negative relationship with negative emotions related to entrepreneurial actions. They also show that PsyCap indirectly enhanced EI. More precisely, students with high PsyCap learned more from the program in terms of perceived skills and knowledge and in turn had a higher EI. Moreover, students with high PsyCap had less entrepreneurial action-related doubt, fear and aversion, which also increased EI. This decrease in negative emotions can be explained notably by what students perceived they had learned from the program. This article concludes with the implications of these findings for future research and practical applications.

Entrepreneurship education has received increasing attention in recent years, from both scholars and practitioners (Barba-Sanchez & Atienzo-Sahuquillo, 2018), and there is growing interest in undertaking and intensifying actions to promote and support the idea of entrepreneurship among students around the globe (Gelard & Saleh, 2011). Many authors have stressed the importance of entrepreneurship education to increase students’ awareness of the possibility of an entrepreneurial career (Fini, Grimaldi, Santoni, & Sobrero, 2011). More precisely, a number of empirical studies have already explored the impact of entrepreneurship education on entrepreneurial intention (EI), showing how it can help create the desire to start new businesses (Adekiya & Ibrahim, 2016). However, other studies found that the effect of entrepreneurship training on EI in university students taking entrepreneurship courses was only moderate (Iglesias-Sánchez, Jambrino-Maldonado, Velasco, & Kokash, 2016), or even negative (Von Graevenitz, Harhoff, & Weber, 2010). These conflicting results raise the question of the psychological mechanisms underlying these differences.

In the field of entrepreneurial cognition, which concerns understanding how entrepreneurs think (Mitchell et al., 2002), some authors have investigated the reasons why individuals start up a business (Shepherd, 2004), examining notably their perceptions of the entrepreneurial skills they have acquired and their emotions regarding entrepreneurial actions (e.g., fear, doubt and aversion). Following the same line, it has been suggested that an entrepreneurship education program is first and foremost a way for students to test their aptitude for an entrepreneurial career (Von Graevenitz et al., 2010), and that entrepreneurial education programs should give students a more realistic view both of themselves and of what it takes to be an entrepreneur (Oosterbeek, van Praag, & Ijsselstein, 2010). Entrepreneurial aptitudes (i.e., the capacity to acquire entrepreneurial competences and skills through training) are related to EI and are thus considered as an antecedent of EI for students following entrepreneurial education programs (Martin, McNally, & Kay, 2013). Previous studies have observed differences in students’ perceptions of the benefits of these courses in terms of developing entrepreneurial skills, influencing their intentions to start a venture; the authors suggest that these differences could be explained by psychological characteristics of the students (Fairlie & Holleran, 2012).

Recent studies have thus investigated psychological characteristics, and more precisely personality traits (e.g., internal locus of control, proactive personality), as antecedents of EI (see the review by Schlaegel & Koenig, 2014). In other words, the trait approach has become one of the major ways of investigating the antecedents of EI (Frese & Gielnik, 2014). However, other authors suggest that studies should focus on psychological resources that can be developed, rather than on traits, which do not change over time (Mitchell et al., 2002). These resources include what the positive psychology movement describes as state-like, such as self-efficacy (“having confidence to take on and put in the necessary effort to succeed at challenging tasks”), hope (“persevering towards goals and when necessary redirecting paths to goals”), optimism (“making a positive attribution about succeeding now and in the future”), and resilience (“when beset by problems and adversity, sustaining and bouncing back and even beyond to attain success”) (Luthans, Youssef, & Avolio, 2007, p. 3). These are the core psychological resources of a construct known in the literature as PsyCap (i.e., Psychological Capital; Luthans et al., 2007).

In line with all these considerations, the aim of the present study is to gain a greater understanding of the psychological and cognitive mechanisms that can enhance the EI of students on entrepreneurship education courses in French universities. To this end, we examined the relationship between the multi-dimensional core construct of positive psychological capital (PsyCap) (Luthans & Youssef, 2004; Luthans et al., 2007) and students’ EI (Luthans et al., 2007).

According to the literature, PsyCap is a psychological resource that could help students to choose an entrepreneurial career (Contreras, De Dreu, & Espinosa, 2017), but no study has yet examined why and how it affects EI. To fill this gap, it is important to understand the key theoretical mechanisms through which PsyCap operates. To identify these cognitive mechanisms, we examined two mediational variables. The first is linked to the benefits of training in terms of perceived learned entrepreneurial skills, in line with findings that this variable is an antecedent of EI (Tsai, Chang, & Peng, 2016). Moreover, certain psychological characteristics can predispose individuals to benefit more from entrepreneurial training than others (Fairlie & Holleran, 2012), and PsyCap has been shown to be positively related to learning empowerment (You, 2016). The second mediational variable concerns the negative emotions associated with entrepreneurial actions (i.e., doubt, fear, aversion). It has been demonstrated, for example, that fear of failure could have a negative influence on EI (Tsai et al., 2016) and that some individuals with high PsyCap manage their emotions better and have more positive emotions than others. Our research thus aims to shed light on the processes leading from PsyCap to EI by examining the role of perceived learned skills and emotions among students following entrepreneurship education programs. While previous research focused on the direct effects of these variables, the present study examines the structural relationships between them, providing a better understanding of the factors that affect students’ EI. This could have theoretical and practical implications, notably regarding the design of effective education programs in line with regional and national policies.

## PsyCap and Entrepreneurial Intentions

This research focuses on the relationship between PsyCap and EI, arguing that individuals’ intentions to start their own business is influenced by four factors that are related to their perceived ability to influence their future, namely self-efficacy, hope, optimism, and resilience. Studies focusing on the relationship between PsyCap and EI remain scarce (Contreras et al., 2017; Sebora & Tantiukoskula, 2011), but indicate that individuals who pursue entrepreneurship as a viable career option tend to have a high level of PsyCap (Sebora & Tantiukoskula, 2011). In line with the research of Magaletta and Oliver (1999, p. 540), we assume that self-efficacy, optimism, and hope are related to the core construct of expectancies, in other words “beliefs that desired outcomes would occur, either due to one’s own efforts or to other factors not under one’s control.” In the same vein, PsyCap is concerned with a change from “who you are” to “who you are becoming,” in other words developing one’s actual self to become the possible self (Avolio & Luthans, 2006).

More specifically, self-efficacy is thought to influence not only the individual’s level of effort and persistence on a specific task but also the choice of activities and behaviors. For example, Chen, Greene, and Crick (1998) found evidence of a positive relationship between entrepreneurial self-efficacy and EI in a sample of business and psychology students.

According to Snyder et al. (1991), hope is a positive motivational state that is based on an interactively derived sense of successful 1) agency (goal-directed energy) and 2) pathways (planning to meet goals). Hope strengthens an individual’s willpower and ability to cope in the face of hurdles. It is thus one of the determining factors in setting goals and devising plans for the future, imagining multiple pathways towards achieving goals, and responding strongly in the event of unexpected occurrences (Peterson & Luthans, 2003).

Optimism is another aspect of PsyCap, defined as an individual’s expectation of positive outcomes or making positive attributions about the likelihood of success in the short or long term (Luthans et al., 2007). According to Magaletta and Oliver (1999, p. 541), the difference between optimism and hope is that the latter concerns the expectancy that positive outcomes are “obtained by the efforts of oneself,” whereas optimism is the expectancy that outcomes are “obtained through others and forces outside the self.” People take the risk of investing money or other resources, even when there are uncertainties, because they expect positive returns on investment (Rigotti, Ryan, & Vaithianathan, 2011). Hence, individuals need to have optimism in order to accept the risks of starting or developing a business venture (Storey, 2011). More broadly, research on entrepreneurs and optimism suggests that it is likely that EI will be associated with the belief that, although the future is uncertain and the path to it bumpy, outcomes will generally end up being positive rather than negative (Puri & Robinson, 2007).

Finally, resilience, which is the ability to go on with life, or to continue living a purposeful life after hardship or adversity (Tedeschi & Calhoun, 2004), has gained recent interest in entrepreneurship research from the viewpoint of how entrepreneurs start new projects after previously failed attempts (Cope, 2011). Being an entrepreneur will involve having to confront hardship because of uncertain situations. Furthermore, apart from the effect on EI of perceptions of desirability and feasibility, the individual’s “propensity to act” on perceived opportunities is also critical (Krueger, Reilly, & Carsrud, 2000). Resilient individuals take action in the face of adversity; they have a higher propensity to act than less resilient individuals, who are easily discouraged by the challenges of a risky environment (Bullough, Renko, & Tamara, 2014).

While these previous studies suggest the importance of the link between these psychological resources and EI, little attention has been paid to these components of the higher order construct of PsyCap (Luthans & Youssef-Morgan, 2017) as an important antecedent of student’s EI. The present research fills this gap by examining the relations between PsyCap and the EI of young students following entrepreneurial education programs.

**H1:** PsyCap is positively related to entrepreneurial intention.

## The Mediational Role of Perceived Learning From the Entrepreneurship Program in the Relationship Between PsyCap and EI

Some studies have revealed differences between students in terms of the perceived benefits gained from entrepreneurship education programs, with some perceiving their entrepreneurial skills to be poor after the training sessions (Othman & Nasrudin, 2016). These findings suggest the importance of examining individual antecedents that could increase students’ perceived entrepreneurial skills and knowledge at the end of their training course. We suggest that the quality of the student learning experience (i.e., perceived acquisition of entrepreneurial skills and knowledge) depends on the individual’s psychological resources (i.e., PsyCap). While there has been little research on PsyCap in academic settings (e.g., You, Kim, & Wang, 2014; You, 2016), findings generally support the positive impact of PsyCap on students’ self-directed learning and learning engagement (You et al., 2014), or learning empowerment (You, 2016). Recently, Liao and Liu (2016) found a link between PsyCap and perceived competence among nursing students, which they postulated could be due to the fact that students with higher PsyCap have the determination and willpower to carry out their internship tasks better, have the capability to deal with obstacles, and have positive attributions (Sun, Zhao, Yang, & Fan, 2012). There is a growing body of empirical evidence that PsyCap has a significant influence on students’ attitudes that is positively related to their academic performance and self-perceived skills (Blumenfeld & Pokay, 1990).

These findings suggest that students’ PsyCap could be an important resource for learning from education programs and could facilitate the development of perceived entrepreneurial skills and knowledge, which would in turn increase their EI. Indeed, perceived capability (i.e., perceived entrepreneurial skills and knowledge) has been demonstrated as a key predictor of intent to start a business (Tsai et al., 2016). As suggested by Souitaris, Zerbinati, and Al-Laham (2007), EI might be influenced by the specific knowledge about entrepreneurship acquired during a learning program. More precisely, learning-derived knowledge leads to more and better entrepreneurial opportunities (Shepherd & DeTienne, 2005) and could also raise students’ EI (Souitaris et al., 2007).

**H2:** The relationship between PsyCap and EI is mediated by perceived learning from the education program.

## The Mediational Role of Negative Emotions Related to Entrepreneurial Actions in the Relationship Between PsyCap and EI

Recognizing the centrality of affect in motivation and decision-making, some studies have examined how this emotional experience influences entrepreneurial decision-making processes (e.g., Li, 2011) and intention (Tsai et al., 2016). More precisely, some authors argue that some action-related emotions in entrepreneurship, such as doubt, fear or aversion, produce hesitancy, promote indecision and encourage procrastination (McMullen & Shepherd, 2006). For example, some studies have found that people are prone to postponing aversive tasks to avoid the unpleasant feelings they provoke (Steel, 2007). Ekore and Okekeocha (2012) also observed that fear of failure discourages university graduates from starting a business, even when the opportunity exists; more generally, this emotion exerts a negative impact on the decision to become self-employed (Arenius & Minniti, 2005). Thus, there is a pervasive tendency to focus on this emotional state and its impact on the intention/decision to start a business, indicating that these negative emotions reduce an individual’s propensity to start a venture (Li, 2011).

Some authors have also demonstrated that certain psychological variables, such as trait self-control, exert a negative and statistically significant effect on action-related fear, aversion, and doubt (i.e., negative emotions) in relation to EI (Van Gelderen, Kautonen, & Fink, 2015). Following this line, we suggest that PsyCap could decrease these negative emotions. According to Van Gelderen et al. (2015), doubt in aspiring entrepreneurs is defined as not knowing how to embark on the start-up process, and as feeling uncertain about the effects and appropriateness of alternative actions. Fear is defined as the experience of anxiety in relation to conducting entrepreneurial activities. Aversion refers to feeling repelled by conducting entrepreneurial activities. From a PsyCap perspective, the common resource between the four capabilities is “positive agent striving” (Avey, Wernsing, & Luthans, 2008), in other words, the desire to act in one’s environment as agent, based on positive personal appraisal of the situation. In line with this view, higher levels of PsyCap have been shown to trigger positive emotions (Snyder et al., 1991).

More precisely, according to the definition of PsyCap, people who are optimistic tend to “dare to dream” and pursue their dreams because they do not fear change, and they also tend to see the positive side of a situation. Hopeful people also experience fewer negative emotions, even when faced with obstacles (Snyder, Ilardi, Michael, & Cheavens, 2000). People with high self-efficacy have a conviction (or confidence) about their abilities to mobilize the motivation, cognitive resources, and courses of action needed to successfully execute a specific task within a given context (Stajkovic & Luthans, 1998). Moreover, resilience, because it is the capacity to bounce back from negative events such as adversity, conflict and failure, is seen as the most important positive resource to manage stressful situations such as an entrepreneurial creation project (Luthans, 2002). In sum, by increasing positive emotions and decreasing negative emotions, PsyCap can help activate more positive cognitive-affective processing system units (e.g., positive expectancies, approach rather than avoidance goals, see Hannah & Luthans, 2008), which can indirectly raise EI. Based on these findings, we made the following hypotheses:

**H3:** The relationship between PsyCap and EI is mediated by negative emotions related to entrepreneurship (action doubt, action fear and action aversion).

## The Mediational Role of Perceived Learning From the Education Program and Negative Emotions Related to Entrepreneurship

What is learnt from the entrepreneurship education program and the negative emotions related to entrepreneurial actions mediate the effect of PsyCap on EI. This mediation is expected to occur because the perceived acquisition of entrepreneurial skills and knowledge may be an effective way to reduce negative emotions related to entrepreneurial actions. In other words, the incentive to start up a business is greater when the entrepreneurs believe that their actions will have achievable outcomes (students perceive that they have acquired knowledge and entrepreneurial skills), notably because this belief reduces avoidance-oriented emotions (action doubt, fear and aversion). In the context of experiential learning, the control-value theory (Pekrun, 2009) postulates that the perceived value and degree of control over an activity or outcome influence the student’s emotional response. In this way, perceived control and value of outcomes form important antecedents of achievement emotions (Pekrun & Perry, 2014). Perceived control refers to how individuals think they can effectively manage a given situation, based on causal expectations and attributions of success and failure (e.g., Weiner, 2007) and on their underlying belief in their competence (e.g., self-concept of ability; Marsh, 1993). It is hypothesized that perceived control positively influences positive emotions, and negatively influences negative emotions (e.g., Niculescu, Tempelaar, Dailey-Hebert, Segers, & Gijselaers, 2015). In other words, in the entrepreneurship context, entrepreneurs who have learned from the education program (i.e., with high levels of perceived acquisition of entrepreneurial knowledge and skills) would have greater perceived control on entrepreneurial actions and consequently feel fewer negative emotions related to them.

**H4:** The perceived learning from the education program in terms of entrepreneurial knowledge and skills (M1) and negative emotions related to entrepreneurial actions (M2) mediate the effect of PsyCap on EI.

## Method

### Data, Setting, and Participants

Data were collected from a sample of 460 French university undergraduates (249 men [54.13%], 209 women [45.43%] and two who did not specify their gender), aged between 18 and 48 years (*M* = 22.45 years; *SD* = 3.14 years). All the participants were at the end of their degree course and were taking an optional 6-month entrepreneurial program (see Supplementary Materials). This program is based on group learning and team competition. The participants were in 298 teams of two to eight members. This entrepreneurship program aims to develop a sense of business, and includes a “taught” component, with different modules (accounting, finance, marketing, management); a “business-planning” component, which includes business plan competitions and advice on developing a specific business idea; and an “interaction with practice” component, which includes talks from practitioners and networking events.

Participants completed this questionnaire on the final day of the course. They were informed about the study’s aims and procedure and were told that the questionnaires were for research purposes only and that their participation was voluntary and anonymous. All participants provided their informed consent to participate in the study. Ethical approval of the study was obtained from the relevant university ethics committee.

### Materials

All multi-item scales were translated from English into French using a four-step procedure: forward translation, assessment, back translation, and assessment based on the criteria of clarity, everyday language, and cultural appropriateness (Presser et al., 2004).

### Psychological Capital

Psychological Capital (PsyCap) was assessed with a reduced version (12 items) of the original 24-item Psychological Capital Questionnaire (Luthans et al., 2007). This 12-item questionnaire (PCQ-12) included three items for self-efficacy, four items for hope, two items for optimism, and three items for resilience. The PCQ-12 has demonstrated acceptable reliability, and its construct validity has been confirmed by several previous studies (Avey, Luthans, & Mhatre, 2008). Items included: “I feel confident contributing to discussions about the team’s strategy” for self-efficacy; “I can think of many ways to reach my current work goals” for hope; “I always look on the bright side of things regarding my job” for optimism; “I can get through difficult times at work because I’ve experienced difficulty before” for resilience. Answers were given on a Likert-type scale ranging from 1 (*strongly disagree*) to 5 (*strongly agree*).

### Perceived Learning From the Entrepreneurship Program

Perceived learning from the entrepreneurship program was measured with the perceptual scale developed by Souitaris et al. (2007). At the end of the course, the students were asked five questions (e.g., “To what extent did the entrepreneurship program enhance your practical management skills in order to start a business) that they answered on a 7-point Likert scale from 1(*not at all*) to 7 (*to a large extent*).

### Negative Emotions

Negative emotions were assessed with eight items in three subscales developed by Van Gelderen et al. (2015): the action doubt subscale (three items), the action fear subscale (two items), and the action aversion subscale (three items). Examples of items are: “It was unclear to me what actions are required to start a business” for action doubt; “The thought of actually taking steps to start my intended business scared me” for action fear; “There were tasks associated with starting my intended business that felt aversive to me” for action aversion. Response categories ranged from 1 (*strongly disagree*) to 7 (*strongly agree*).

### Entrepreneurial Intentions

Entrepreneurial intentions (EI) were measured with a 2-item scale drawn from Lee, Wong, Foo, and Leung (2011). Respondents indicated their level of agreement with each statement (“If I had the opportunity, I would start my own company” and “I have always wanted to work for myself (i.e., be self-employed)”) on a Likert-type scale from 1 (*strongly disagree*) to 7 (*strongly agree*). Lee et al. (2011) reported adequate reliability (α = 0.72) and convergent validity with an established measure of EIs (Kolvereid, 1996, *r* = .79, *p* < .01). Tolentino, Sedoglavich, Lu, Garcia, and Restubog (2014) also reported adequate reliability (α = 0.85).

### Control Variable

Due to the gender differences observed in entrepreneurship (Ahl, 2006; Herrington & Kew, 2017), gender was measured as a dummy variable, with men coded as 0 and women coded as 1, and was used as a control variable for EI.

### Statistical Analysis

See Supplementary Materials.

## Results

### Preliminary Analyses

First, the age distribution of the sample was examined (see Supplementary Materials), and eight participants aged over 33 years and three participants who did not specify their age were excluded. Hence, we performed the analyses with a sample of 449 individuals.

Secondly, Shapiro-Wilk tests revealed that all study variables were non-normally distributed (see Table 1). Therefore, the MLR estimation was used in structural equation models.

**Table 1:** Results of Shapiro-Wilk Tests Conducted on the Study Variables (N = 449)

Variable	Statistic	*df*	*p*
Psychological capital	.98	449	< .001
Learning from the program	.97	449	< .001
Negative emotions	.99	449	.02
Entrepreneurial intention	.95	449	< .001

Examination of the correlations between the variables used in this survey (see Table 2) provided support for our hypotheses, as the relationships between all study variables were statistically significant (*p* < .001). Moreover, all the scales demonstrated good internal consistency, with Cronbach’s α coefficients higher than 0.77 (see Table 2), and confirmatory factor analysis yielding a satisfactory fit: SBχ^2^ (311) = 544.36, *p* < .001, TLI = 0.92, CFI = 0.93, RMSEA = 0.05 and SRMR = 0.04. These findings support the validity and reliability of the tools used in this research.

**Table 2:** Cronbach’s Alphas, Means, Standard Deviations and Pearson Correlations Among the Study Variables (N = 449)

Variable	α	*M*	*SD*	1	2	3
1. Psychological capital	0.85	3.95	0.53			
2. Learning from the program	0.82	5.45	0.94	.38***		
3. Negative emotions	0.84	3.64	1.09	−.43***	-.35***	
4. Entrepreneurial intention	0.77	4.93	1.45	.31***	.34***	−.37***

*Note. SD* = standard deviation.

***p < .001.

### Main Analyses

**Figure 1:**
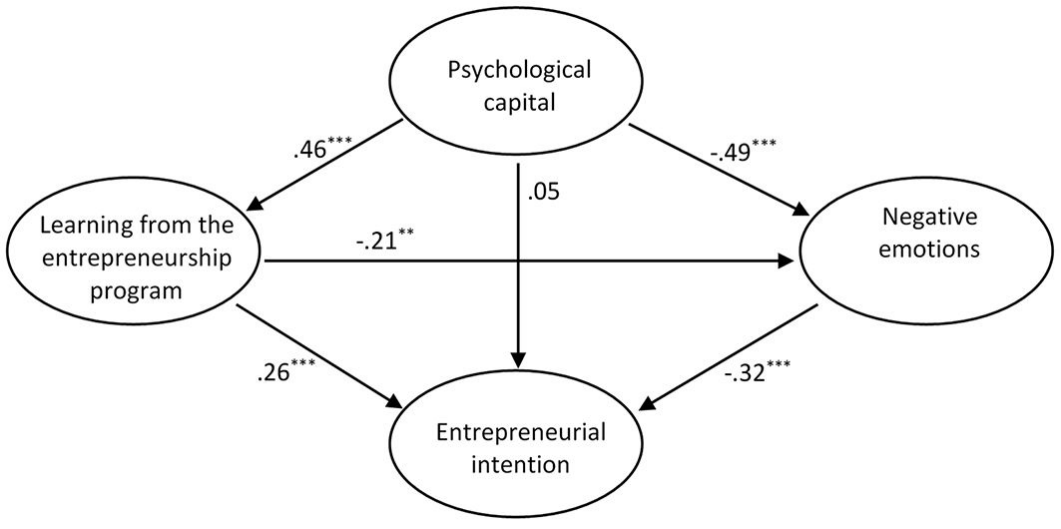
Results of the Hypothesized Mediational Model Using SEM *Note*. All variables are latent variables. Standardized parameter estimates of direct effects obtained with the MLR method are displayed. **p < .01. ***p < .001.

Our hypothesized model illustrated in Figure 1 yielded a satisfactory fit to the data: SBχ^2^ (337) = 633.58, *p* < .001, TLI = 0.90, CFI = 0.91, RMSEA = 0.05 and SRMR = 0.06.

For clarity, gender is not represented but is included in the model as a control variable for Entrepreneurial intention.

The results of SEM path analysis did not support Hypothesis H1: there was no significant direct effect of PsyCap on EI (β = 0.05, *p* = .56) but its indirect effects were significant, as described below.

Bootstrap analyses revealed that PsyCap had a significant positive indirect effect on EI (β = 0.31; 95% CI [0.22, 0.43]). The indirect effect of PsyCap on EI was significant, whereas the direct effect was insignificant. Thus, the relationship between PsyCap and EI was fully mediated.

More precisely, the results indicated that the three indirect effects corresponding to our hypotheses H2, H3 and H4 (i.e., PsyCap → learning from the program → EI, PsyCap → negative emotions → EI and PsyCap → learning from the program → negative emotions → EI, respectively) were significant (see Table 3). Therefore, Hypotheses H2, H3 and H4 were supported.

**Table 3:** Estimated Standardized Indirect Effects and 95% Confidence Intervals for Testing H2, H3 and H4, Using Bias-Corrected Bootstrapping

Mediation Path	β	95% CI	
		*LL*	*UL*
**H2:** PsyCap → learning from the program → EI	0.12	0.06	0.20
**H3:** PsyCap → negative emotions → EI	0.16	0.08	0.28
**H4:** PsyCap → learning from the program → negative emotions → EI	0.03	0.01	0.07

*Note*. EI = entrepreneurial intention. For this analysis, 5,000 bootstrap samples were generated.

## Discussion

In entrepreneurship research, EI is considered to be a significant predictor of subsequent business creation behavior (Kautonen, Van Gelderen, & Tornikoski, 2013), which is a crucial issue for economic development in all the countries of the world. Accordingly, there are increasing efforts to foster entrepreneurship through education and training in universities. To understand how EI can be increased among students taking part in entrepreneurship programs, this study focused on the individual antecedents of EI on which it could be possible to act. More precisely, we investigated the relationships between PsyCap, perceived learning from the entrepreneurship program, negative emotions and EI. To date, there is insufficient empirical evidence to establish any meaningful relationship between PsyCap and EI among students in these education entrepreneurship programs and to identify the mediational variables that could explain this relationship. Moreover, the results about the impact of these programs on EI are inconsistent, and there is little knowledge about the individual antecedents of students’ EI, notably concerning the specific variable of PsyCap, for which there have been few studies in this area.

Our results showed that students’ PsyCap had a significant positive relationship with perception of learning from the program and a significant negative relationship with negative emotions related to entrepreneurial actions. They also underlined that PsyCap indirectly enhanced EI. More precisely, students with high PsyCap learned more from the program in terms of perceived skills and knowledge and in turn had a higher EI. Moreover, students with high PsyCap had less entrepreneurial action-related doubt, fear and aversion, which also increased EI. This decrease in negative emotions can be explained notably by what students perceived they had learned from the program.

Several conclusions can be drawn from our findings. First, the results are consistent with previous research about the influence of PsyCap on positive entrepreneurial outcomes more generally (Baluku, Kikooma, Bantu, & Otto, 2018), and with studies revealing the benefits of PsyCap in the academic domain (You, 2016) and on positive emotions (Avey, Wernsing, & Luthans, 2008). Our study extended the conclusions of those studies by including all these variables in an integrative model in a sample of students participating in an entrepreneurship program. Moreover, as our findings support the PsyCap literature and confirm the benefits of PsyCap in entrepreneurship education, they enhance our understanding of the effects of PsyCap.

This study also addresses an issue raised by previous research, namely that entrepreneurship studies should take a state-rather than a trait-based approach. Increasing students’ intent to start a business is an important issue, and enhancing their PsyCap adds a new dimension.

From a practical point of view, this research suggests that entrepreneurship education can be enhanced by focusing on the components of PsyCap (i.e., self-efficacy, hope, optimism, and resilience), and encouraging students to build on these dimensions. Indeed, in addition to its scholarly contribution, this study can help raise institutional awareness of the importance of students’ PsyCap, by showing that it is a fundamental resource for developing the intention to create a business. In line with research showing that PsyCap is a malleable, open-to-development individual difference variable (e.g., Luthans, Avey, & Patera, 2008), our results suggest that the assessment, development, and management of PsyCap should be included in the entrepreneurship program. For example, Luthans and his colleagues (2008, 2010, 2012) developed a specialized psychological capital intervention (PCI) model, designed to develop PsyCap in the workplace. They showed that psychological capital development led to significant improvement in individual and organizational performance.

This PCI model could be used in entrepreneurial education to enhance students’ self-efficacy, optimism, hope, and resilience, as well as their overall PsyCap (Dello Russo & Stoykova, 2015; Luthans et al., 2007, 2008). For example, to develop hope, participants could be asked to identify key goals they wish to achieve during the session. The instructor could then explain the need for 1) concrete end points to measure success; 2) an approach (rather than an avoidance) framework enabling them to work toward goal accomplishment; and 3) a “stepping” method to identify subgoals in order to reap the benefits of even small achievements. For self-efficacy, participants should be given the opportunity to experience success through positive “self-talk” and visualization to gain “imaginal” task mastery experience. This PsyCap training intervention could also help develop students’ resilience. For example, participants could be asked to identify recent personal minor or major setbacks and their immediate reactions to these situations. Their coping strategies could then be compared with examples of an ideal resilient process for dealing with setbacks, based on the broaden-and-build positivity approach advocated by Fredrickson (2001).

While a number of studies have reported that even short training programs are effective in increasing PsyCap (Luthans et al., 2008, 2012), more research is needed to confirm our results, before developing PsyCap training programs and specific instructional design guidelines for embedding PsyCap in entrepreneurship education programs.

The present study highlights the crucial role of PsyCap on EI and suggests that training programs should focus on its development in order to optimize the learning benefits and the emotions related to entrepreneurship. Overall, in view of the positive outcomes of PsyCap, it seems particularly important to focus on this psychological resource.

### Limitations and Future Research

This study has several limitations that should be addressed in future research. Due to its cross-sectional design, it is difficult to generalize the findings. Further studies are therefore needed with other samples of students, particularly in other countries, as research has shown that cultural dimensions influence students’ career decisions through social norms, valuations and practices (Ozarally & Rivenburgh, 2016). Indeed, there are consistent findings of cross-cultural differences in the desire to become an entrepreneur (Flores, Robitschek, Celebi, Andersen, & Hoang, 2010), notably among students (Lüthje & Franke, 2003). For example, U.S. students consider their national culture more “entrepreneurially supportive” than Turkish students (Ozarally & Rivenburgh, 2016), underlining the need to control cultural dimensions in future multi-cultural research.

More research is needed to confirm these results in other entrepreneurship courses and programs. Our findings also raise the issue of whether entrepreneurship education can have broader repercussions on both the psychological and the cognitive development of young people (Groves, Vance, & Choi, 2011). Moreover, future research should investigate other mediational variables between PsyCap and EI, such as stress management (Baron, Franklin, & Hmieleski, 2016), which is a crucial skill for entrepreneurs. It would also be interesting to investigate sleep as a mediational variable; PsyCap has already been observed to be related to fatigue (Hystad & Eid, 2016), and it has recently been postulated that sleep could be related to EIs, not only by influencing perceptions of the desirability and feasibility of pursuing existing entrepreneurial ideas, but also by heightening the propensity to act (Gunia, Gish, & Mensmann, 2021). Longitudinal studies could also provide further information about the links between the relationships between PsyCap, learning from the program, emotions, and EI or other positive constructs related to entrepreneurship education programs.

While this study has a number of limitations, it highlights the positive relationship between PsyCap and EI in students following entrepreneurship education programs. It suggests avenues for future research in this area and contributes to our knowledge of ways to train tomorrow’s entrepreneurs.

## Supplementary Materials

For this article the following Supplementary Materials are available via PsychArchives (for access see Index of Supplementary Materials below).

- A figure displaying the course outline of the entrepreneurial training.
- A frequency histogram displaying the age distribution in the sample of the study.
- The statistical procedure used to the test the hypothesized model using SEM.
